# High-throughput immunogenetic typing of koalas suggests possible link between MHC alleles and cancers

**DOI:** 10.1007/s00251-020-01181-7

**Published:** 2020-10-20

**Authors:** Bonnie L. Quigley, Galit Tzipori, Karen Nilsson, Peter Timms

**Affiliations:** 1 Genecology Research Centre, University of the Sunshine Coast, 90 Sippy Downs Drive, Sippy Downs, QLD 4556 Australia; 2 Lone Pine Koala Sanctuary, Fig Tree Pocket, QLD Australia

**Keywords:** Koala, *Phascolarctos cinereus*, MHC, Immunogenetic profiling

## Abstract

Characterizing the allelic diversity within major histocompatibility complex (MHC) genes is an important way of determining the potential genetic resilience of a population to infectious and ecological pressures. For the koala (*Phascolarctos cinereus*), endemic diseases, anthropogenic factors and climate change are all placing increased pressure on this vulnerable marsupial. To increase the ability of researchers to study MHC genetics in koalas, this study developed and tested a high-throughput immunogenetic profiling methodology for targeting MHC class I UA and UC genes and MHC class II DAB, DBB, DCB and DMB genes in a population of 82 captive koalas. This approach was validated by comparing the determined allelic profiles from 36 koala family units (18 dam-sire-joey units and 18 parent-joey pairs), finding 96% overall congruence within family profiles. Cancers are a significant cause of morbidity in koalas and the risk factors remain undetermined. Our analysis of this captive population revealed several novel MHC alleles, including a potential link between the DBB*03 allele and a risk of developing cancer. This method offers a reliable, high-throughput protocol for expanded study into koala immunogenetics.

Major histocompatibility complex (MHC) genes play a critical role in the immune system. MHC molecules present antigens from either intracellular threats (such as viruses and cancerous proteins, via class I molecules) or phagocytosed antigens (such as bacteria and parasites, via class II molecules) to T lymphocytes to initiate an adaptive immune response (Punt et al. 2018). In vertebrates, MHC allelic variation in a population has been linked to biological traits from immune recognition and susceptibility to infectious and autoimmune diseases and to ecological success with mating preferences and pregnancy outcomes (Sommer 2005). For the last remaining member of the family Phascolarctidae, the koala (*Phascolarctos cinereus*), survival against both endemic disease (from *Chlamydia pecorum* and potentially koala retrovirus) and population fragmentation/genetic bottlenecking has reached a crisis point (Australia 2011; Hemming et al. 2018). This has recently led to an increased focus on studying of MHC genetic loci in koalas to understand their potential genetic resilience in the face of these ecological pressures.

There are 23 MHC class I and 23 MHC class II genes and pseudogenes annotated in the koala genome (Johnson et al. 2018). Detailed investigation into class I genes determined that 11 of these genes are actively transcribed in the koala, with three genes ubiquitously expressed as classical class Ia genes (Phci-UA, UB and UC) and eight genes with tissue restricted expressions as nonclassical class Ib genes (Phci-UD, UE, UF, UG, UH, UI, UJ and UK) (Cheng et al. 2018). All of the expressed MHC class I genes appear to be present as single copy genes in the genome (Cheng et al. 2018; Johnson et al. 2018). Within the MHC class II gene family, four class II subfamilies are recognized, consisting of alpha and beta subunits of DA, DB, DC and DM (Abts et al. 2018; Johnson et al. 2018; Lau et al. 2013). Studies investigating the allelic diversity of class II DA and DB genes have found that the beta subfamilies (DAB and DBB) contain more allelic diversity than the alpha subfamilies (DAA and DAA) (Lau et al. 2013). In addition, genome analysis and diversity studies indicate that DAB and DBB genes are present as three distinct loci in the genome, while DCB and DMB genes are present as single copy genes (Johnson et al. 2018; Lau et al. 2013).

Several techniques have been used to identify the allelic diversity of MHC class I and II genes in wild and captive koala populations. Initial studies focused on class II DA and DB genes and utilized single-strand conformation polymorphism (OSCP) analysis (Lau et al. 2014a, 2014b, 2013). OSCP analysis involves PCR amplification of the target loci, lambda exonuclease digestion to remove the forward amplicon stand and acrylamide gel electrophoresis of the reverse amplicon strand to generate a banding pattern and excising of individual bands for direct sequencing or cloning and sequencing to determine allele sequences (Lau et al. 2013). While this approach has the advantage of ensuring all alleles within an individual are detected (via each allele’s unique banding position in the gel), this method is very labour intensive and low throughput. Later studies investigating class I UA, UB and UC genes or class II DA and DB genes opted for the directly cloning and sequencing of PCR amplicons from the target loci (Cheng et al. 2018; Quigley et al. 2018). While this approach improved throughput, it could not guarantee every allele for a tested gene was detected in the set of sequenced clones. Most recently, direct Illumina sequencing of PCR amplicons from the target loci was attempted for a range of class I and II gene loci (Abts et al. 2018). This approach allowed for higher throughput processing and greatly increased confidence that all the allelic diversity within a koala would be detected; however, sequence processing challenges related to amplicon size and multiple genes per loci limited the number of targets that generated reportable data. The field of koala MHC immunogenetics needs a comprehensive approach that combines the advantages of previous studies into a single, reliable, high-throughput technique. That is what this study achieved (Fig. 1).

**Fig. 1:**
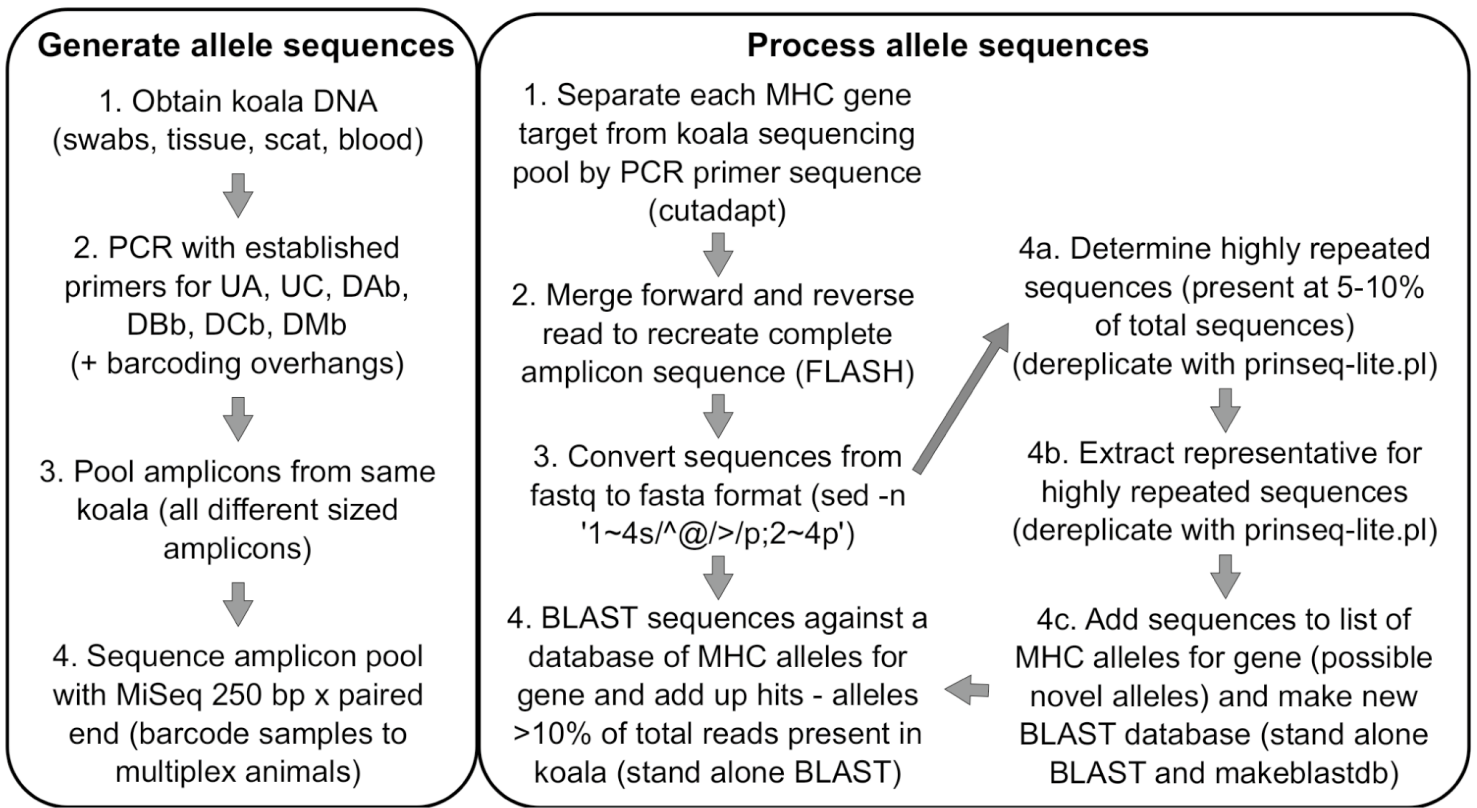
Flowchart of high-throughput MHC allele determination method in koalas. The left panel summarizes the steps from sample acquisition to sequence generation while the right panel summarizes sequence processing to allele assignment (with example programs/commands necessary to complete each step given in parentheses)

In our current study, the immunogenetic profile of 82 captive koalas was generated in a high-throughput fashion. Blood samples were collected from koalas from Lone Pine Koala Sanctuary (Brisbane, Queensland, Australia) as part of routine health monitoring. DNA was extracted using the DNeasy Blood & Tissue kit (Qiagen) as per manufacturer’s instructions. Established PCR primers that target the receptor binding grove (exon 2 region) of class I UA and UC genes and class II DAB, DBB, DCB and DMB genes (Table 1) (Abts et al. 2018; Cheng et al. 2018; Lau et al. 2013) were used to generate loci-specific amplicons between 200–397 bp. To increase throughput and reduce costs, adaptor sequences were added to the 5′ end of each primer to allow for multiplex barcoding of koala samples for sequencing. The six MHC gene target amplicons from each koala were pooled for barcoding (generating one barcoded sample per koala) and all 82 koala samples were pooled for sequencing on a single MiSeq 250 bp paired end Illumina run (Ramaciotti Centre for Genomics, Sydney) (Fig. 1).

**Table 1:** PCR primers used to identify MHC alleles

MHC class	Gene target	Primer sequence^a^	Amplicon size (bp)	Reference
Class I	UA	MHCI_UA_F: ACCCCTGACCCTGCCGTGTC	313	Cheng et al. (2018)
		MHCI_UA_R: CACCGCCTTCGCTCTGGTTGA		
	UC	MHCI_UC_F: AAGGTCTCCAATGTTTCCGACTCA	397	Cheng et al. (2018)
		MHCI_UC_R: TCTCGCGCTAAGGCCATACC		
Class II	DAb	MHCII_DAb_F: ATGCCCCAAAGCACTTCAC	271	Lau et al. (2013)
		MHCII_DAb_R: CGCACTRAGAAGGGCTCA		
	DBb	MHCII_DBb_F: AGGGACATCCCAGAGGATTTCG	282	Lau et al. (2013)
		MHCII_DBb_R: TCTTCTGTCCACCGCGAAGG		
	DCb	MHCII_DCb_F: GGTGAGGTCTGAGTGTCACA	200	Abts et al. (2018)
		MHCII_DCb_R: CATTCACTATGGACCTTGCAGT		
	DMb	MHCII_DMb_F: CATGTGGAGAGTGGCTGTATG	266	Abts et al. (2018)
		MHCII_DMb_R: GTCCTTTGGGTCAACGCTC		

^a^For Illumina MiSeq sequencing, adaptor sequences were added to the 5′ ends of each primer to allow for multiplex barcoding: forward adaptor: TCGTCGGCAGCGTCAGATGTGTATAAGAGACAG; reverse adaptor: GTCTCGTGGGCTCGGAGATGTGTATAAGAGACAG

To deconvolute the raw sequencing results into MHC alleles present per koala, the sequences obtained from each koala were sorted, merged into complete amplicon sequences, processed to extract highly repeated sequences and identified against a database of known koala alleles (Fig. 1). Sequence files from each koala were first separated into individual target gene files based on the PCR primer sequence for each target gene, trimmed to remove the primers sequences and culled to remove any reads shorter than 150 bp using the program cutadapt (Martin 2011). Next, paired forward and reverse reads were merged to reassemble the complete amplicon sequence using the program FLASH (Magoc and Salzberg 2011). The sequence data was then converted from Fastq to Fasta format using the standard unix ‘sed’ command. Finally, sequences were BLAST searched against a list of known koala MHC alleles using stand-alone BLAST (Altschul et al. 1990; Camacho et al. 2009). For each gene target, reference alleles that represented more than 10% of the total sequence reads for that gene were considered present in the koala. To detect novel MHC alleles not represented in the reference list, sequence files were separately tested for highly repetitive sequences with the program prinseq (Schmieder and Edwards 2011) and novel sequences were added to the reference list (Fig. 1).

Using this high-throughput method, the allelic diversity of six MHC genes was determined for all 82 test koalas (Fig. 2). Overall, this population contained seven UA alleles (six novel), five UC alleles (three novel), 10 DAB alleles (one novel), eight DBB alleles, three DCB alleles (all novel) and four DMB alleles (all novel) (Fig. 2). Within these alleles, the expected range of 1 to 2 alleles per koala was retrieved from the single genome copy genes UC, DCB and DMC and 1 to 6 alleles per koala were retrieved from the three genome copy genes DAB and DBB. Interestingly, between 1 and 3 alleles per koala were retrieved from UA (a single copy gene). Sequence comparison revealed that the detected UA alleles designated UA*08:01 and UA*09:01 were identical to the previously published UB alleles UB*04:01 and UB*03:01, respectively (Fig. 2). This suggested that the UA PCR primer set was amplifying both UA and UB alleles, and both gene loci are represented in the UA allele results. Phylogenetic analysis of class I sequences supported the fact that UA and UB alleles are closely related, preventing segregation of alleles into UA or UB gene origin (Fig. 2a).

**Fig. 2:**
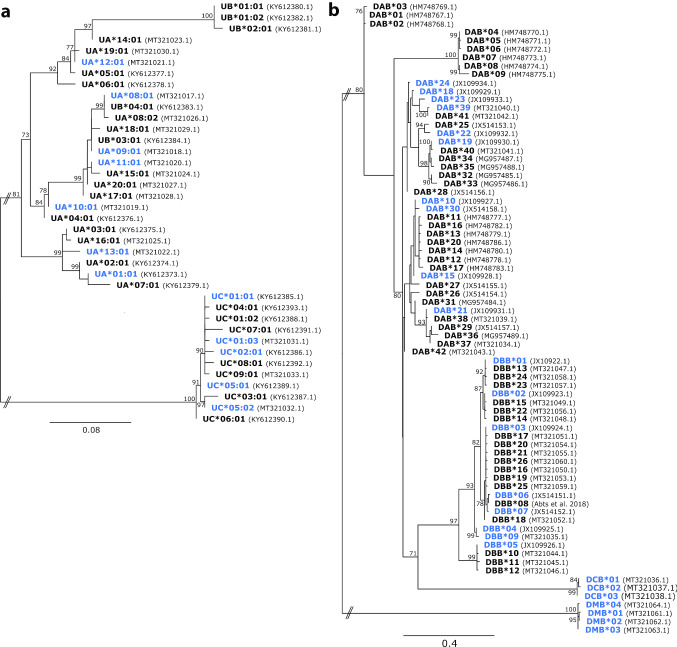
Phylogenetic relationships of known koala MHC gene alleles from class I (**a**) and class II (**b**). These maximum likelihood phylogenetic trees were generated using DNA sequences aligned with mafft (Katoh et al. 2002) before ModelFinder determined the best fit model (HKY + F + G4 for (**a**); TIMe + G4 for (**b**) (Kalyaanamoorthy et al. 2017)) and IQ-TREE (Nguyen et al. 2015) and UFBoot2 (Hoang et al. 2018) constructed the tree with 1000 bootstrap replicates. Only bootstrap values above 70 are shown. Alleles highlighted in blue were detected in this study. The accession number for each allele is presented in parenthesises after the allele name

Within the captive koala study group, there were 18 family units (dam-sire-joey) and an additional 18 parent-offspring pairs (either dam-joey or sire-joey). This allowed for a detailed evaluation of the accuracy of this high-throughput koala immunogenetic approach (Tables 2 and 3). Knowing that MHC alleles must follow Mendel’s law of segregation of genes (offspring must inherit an allele from each parent) and that the genetics of an offspring should be composed of the genetics of their parents, the accuracy of allele assignment within the family groups was determined. Examining all 36 family units, there was 100% congruency between the parent(s)/joey genetic profiles for UA, DCB and DMB loci, 94% (34/36) congruency in DAB and DBB profiles and 86% (31/36) congruncy in UC profiles (Tables 2 and 3). Within these 216 comparisons, the nine inconsistent cases involved five cases where the joey was missing an allele from either their dam or sire (four UC, one DBB), three cases where the joey possessed an allele neither parent possessed (two DAB, one DBB) and one case where the joey did not have an allele from either parent (UC). Overall, these minor discrepancies resulted in this method having an overall congruency rate of 96%.

**Table 2:** Immunogenetic profiles of dam-sire-joey family groups

Family	Relationship	Name	UA	UC	DAB	DBB	DCB	DMB
1	Dam	Jazz	08:01; 10:01; 11:01	01:01	10; 15; 19; 21	02; 05	01; 03	02
	Sire	Barney	08:01; 11:01	01:03	10; 15; 19; 21	02; 05	01	01
	Joey	Angelica	08:01; 10:01; 11:01	01:01; 01:03	10; 15; 19; 21	02; 05	01	01; 02
2	Dam	Rusa	08:01; 09:01; 11:01	*01:01*	15; 19; 21	02; 04	01; 02	02; 03
	Sire	Keeley	08:01; 09:01	*05:01*	15; 19; 21	02; 04	01; 02	02; 04
	Joey	Myrtle	08:01; 09:01; 11:01	*02:01*	15; 19; 21	04	01; 02	02; 03
3	Dam	Jubilee	08:01; 09:01; 11:01	01:01; 05:01	10; 19; 21; 30	01; 03; 04	01; 03	02; 03
	Sire	Byron	10:01; 13:01	01:01; 05:02	15; 19; 21	01; 02	01	02; 03
	Joey	Kia	08:01;10:01; 11:01	01:01	15; 19; 21; 30	02; 03	01	02; 03
4	Dam	Elata	01:01	*01:01*	10; 19; 21	02; 04; 05; 09	03	01; 02
	Sire	Byron	10:01; 13:01	01:01; 05:02	15; 19; 21	01; 02	01	02; 03
	Joey	Sargent	01:01; 13:01	*05:02*	10; 15; 19; 21	01; 04	01; 03	02; 03
5	Dam	Sinammon	08:01; 10:01; 11:01	05:02	10; 15; 19; 21	01	01	03
	Sire	Fingal	08:01; 11:01; 13:01	01:01	10; 15; 19; 21	02; 03; 05	01	01; 04
	Joey	Milton	08:01; 10:01; 13:01	01:01; 05:02	10; 19; 21	01; 03	01	01; 03
6	Dam	Minx	08:01; 11:01	05:02	15; 19; 21; 22; 24; 39	04; 06; 07	01	01; 02
	Sire	Byron	10:01; 13:01	01:01; 05:02	15; 19; 21	01; 02	01	02; 03
	Joey	Tully	08:01: 10:01; 11:01	5:02	15; 19; 21	01; 06; 07	01	02; 03
7	Dam	Halle	01:01; 10:01	01:01	15; 19; 21	02; 05	01; 03	02
	Sire	Keeley	08:01; 09:01	05:01	15; 19; 21	02; 04	01; 02	02; 04
	Joey	Jester	01:01; 08:01; 09:01	01:01; 05:01	15; 19; 21	02; 05	02; 03	02; 04
8	Dam	Kirra	08:01; 09:01; 13:01	01:01	10; 15; 19; 21	02; 03	01	01; 02
	Sire	Strudel	01:01; 12:01	01:01; 01:03	10; 19; 21; 23	02; 03	01	01
	Joey	Esmeralda	12:01; 13:01	01:01	15; 19; 21; 23	02; 03; *04*	01	01; 02
9	Dam	Dama	08:01; 11:01	01:03	10; 15; 19; 21	02; 05	01	01
	Sire	Strudel	01:01; 12:01	01:01; 01:03	10; 19; 21; 23	02; 03	01	01
	Joey	Feenie	08:01; 11:01; 12:01	01:03	15; 19; 21; 23	02; 05	01	01
10	Dam	Victory	08:01; 11:01; 12:01	01:01; 05:01	15; 19; 21; 30	02; 06; 07	01	02; 04
	Sire	Aster	08:01; 10:01; 11:01	01:01	15; 19; 21; 23	02; 04	01; 02	03; 04
	Joey	Nat	08:01; 11:01	01:01	15; 19; 21	02; 04	01; 02	02; 03
11	Dam	Rookie	08:01; 09:01; 12:01	01:01	10; 15; 19; 21	02; 04; 05	02; 03	01; 03
	Sire	Strudel	01:01; 12:01	01:01; 01:03	10; 19; 21; 23	02; 03	01	01
	Joey	Davis	01:01; 08:01; 09:01	01:01; 01:03	10; 15; 19; 21	02; 03; 04; 05	01; 02	01; 03
12	Dam	Rookie	08:01; 09:01; 12:01	01:01	10; 15; 19; 21	02; 04; 05	02; 03	01; 03
	Sire	Barney	08:01; 11:01	*01:03*	10; 15; 19; 21	02; 05	01	01
	Joey	Drew	08:01; 11:01; 12:01	*01:01*	10; 19; 21; *22; 24; 39*	02; 04; 05	01; 03	01
13	Dam	Guppy	08:01; 09:01; 10:01	01:01	10; 19; 21; 22; 24; 39	02; 04	01; 03	01; 02
	Sire	Yeti	01:01; 08:01; 11:01	01:01	15; 19; 21; 23	01; 03	01	01; 02
	Joey	Urchin	01:01; 08:01; 09:01	01:01	10; 19; 21; 23	03; 04	01	01
14	Dam	Zap	01:01; 10:01	01:01	10; 15; 19; 21	02; 05	01; 03	02; 03
	Sire	Byron	10:01; 13:01	01:01; 05:02	15; 19; 21	01; 02	01	02; 03
	Joey	Hamlet	10:01; 13:01	01:01	15; 19; 21	02; 05	01; 03	02
15	Dam	Kirra	08:01; 09:01; 13:01	01:01	10; 15; 19; 21	02; 03	01	01; 02
	Sire	Yeti	01:01; 08:01; 11:01	01:01	15; 19; 21; 23	01; 03	01	01; 02
	Joey	Merlin	08:01; 09:01; 11:01	01:01	10; 15; 19; 21	01; 03	01	01; 02
16	Dam	Crumble	10:01; 12:01	05:01	10; 19; 21	04; 06; 07	01; 03	01; 04
	Sire	Wisely	01:01; 12:01	01:01; 01:03	15; 19; 21	02; 05	01	02
	Joey	Waffle	01:01; 10:01	01:01; 05:01	10; 15; *18*; 19; 21	02; 06; 07	01	02; 04
17	Dam	Zap	01:01; 10:01	01:01	10; 15; 19; 21	02; 05	01; 03	02; 03
	Sire	Byron	10:01; 13:01	01:01; 05:02	15; 19; 21	01; 02	01	02; 03
	Joey	Cordelia	10:01	01:01	15; 19; 21	02	01; 03	02
18	Dam	Minx	08:01; 11:01	05:02	15; 19; 21; 22; 24; 39	04; 06; 07	01	01; 02
	Sire	O'Malley	08:01; 10:01; 11:01	01:01; 05:02	15; 19; 21; 30	01; 03	01	03
	Joey	Archer	08:01; 10:01; 11:01	01:01; 05:02	15; 19; 21; 30	03; 06; 07	01	02; 03

Italicized entries are alleles incongruent with Mendelian laws of genetics

**Table 3:** Immunogenetic profiles of parent-joey family pairs

Family	Relationship	Name	UA	UC	DAB	DBB	DCB	DMB
19	Dam	Elata	01:01	01:01	10; 19; 21	02; 04; 05; 09	03	01; 02
	Joey	Major	01:01; 08:01; 11:01	01:01	10; 19; 21; 30	03; 04	01; 03	02; 03
20	Dam	Minx	08:01; 11:01	*05:02*	15; 19; 21; 22; 24; 39	*04; 06; 07*	01	01; 02
	Joey	Barney	08:01; 11:01	*01:03*	10; 15; 19; 21	*02; 05*	01	01
21	Dam	Dama	08:01; 11:01	01:03	10; 15; 19; 21	02; 05	01	01
	Joey	Sprocket	08:01; 09:01; 11:01	01:01; 01:03	10; 15; 19; 21	02; 03; 05	01	01; 02
22	Dam	Mooloolah	08:01; 11:01; 13:01	01:01	10; 15; 19; 21	02; 03	01	01; 02
	Joey	Clifton	08:01; 09:01; 13:01	01:01; 05:02	10; 15; 19; 21	01; 02	01	02; 03
23	Dam	Mooloolah	08:01; 11:01; 13:01	01:01	10; 15; 19; 21	02; 03	01	01; 02
	Joey	Byron	10:01; 13:01	01:01; 05:02	15; 19; 21	01; 02	01	02; 03
24	Dam	Liana	10:01; 12:01	01:01; 01:03	21; 23	02; 03; 05	01	01; 02
	Joey	Daiquiri	08:01; 09:01; 10:01	01:01	15; 19; 21; 23	03	01; 03	01; 02
25	Dam	Elata	01:01	*01:01*	10; 19; 21	02; 04; 05; 09	03	01; 02
	Joey	Virginea	01:01; 10:01	*02:01*	10; 15; 19; 21	04	02; 03	02; 03
26	Dam	Mooloolah	08:01; 11:01; 13:01	01:01	10; 15; 19; 21	02; 03	01	01; 02
	Joey	Jervis	08:01; 11:01; 13:01	01:01	10; 19; 21; 30	03; 04	01	01
27	Dam	Rusa	08:01; 09:01; 11:01	01:01	15; 19; 21	02; 04	01; 02	02; 03
	Joey	Aster	08:01; 10:01; 11:01	01:01	15; 19; 21; 23	02; 04	01; 02	03; 04
28	Dam	Dama	08:01; 11:01	01:03	10; 15; 19; 21	02; 05	01	01
	Joey	Fraggle	01:01; 08:01; 11:01	01:01; 01:03	10; 15; 19; 21	01; 02; 05	01	01; 02
29	Dam	Mooloolah	08:01; 11:01; 13:01	01:01	10; 15; 19; 21	02; 03	01	01; 02
	Joey	Fingal	08:01; 11:01; 13:01	01:01	10; 15; 19; 21	02; 03; 05	01	01; 04
30	Dam	Elata	01:01	01:01	10; 19; 21	02; 04; 05; 09	03	01; 02
	Joey	Pellita	01:01; 12:01	01:01	10; 19; 21; 23	03; 04	01; 03	01; 02
31	Dam	Mooloolah	08:01; 11:01; 13:01	01:01	10; 15; 19; 21	02; 03	01	01; 02
	Joey	Kirra	08:01; 09:01; 13:01	01:01	10; 15; 19; 21	02; 03	01	01; 02
32	Dam	Rusa	08:01; 09:01; 11:01	01:01	15; 19; 21	02; 04	01; 02	02; 03
	Joey	Bkley	08:01; 09:01; 11:01	01:01	15; 19; 21	03; 04	01; 02	01; 03
33	Sire	Byron	10:01; 13:01	01:01; 05:02	15; 19; 21	01; 02	01	02; 03
	Joey	Tango	08:01; 10:01; 11:01	01:01	10; 15; 19; 21	02	01	02
34	Sire	Rory	10:01	02:01	10; 19; 21; 22; 24; 39	04	01; 03	01; 02
	Joey	Hermit	08:01; 10:01; 11:01	02:01	10; 19; 21	04	03	01; 02
35	Sire	Orinoco	08:01; 10:01; 11:01	01:01; 05:02	15; 19; 21	01; 02	01	03
	Joey	Ficus	08:01; 10:01; 11:01	01:01; 05:02	15; 19; 21	01; 02	01	02; 03
36	Sire	Fingal	08:01; 11:01; 13:01	01:01	10; 15; 19; 21	02; 03; 05	01	01; 04
	Joey	Claret	08:01; 11:01; 13:01	01:01	15; 19; 21; 23	02; 04; 05	01; 03	02; 04

Italicized entries are alleles incongruent with Mendelian laws of genetics

To examine the immunogenetic diversity within this koala captive population, MHC haplotypes were clustered in R (R_Core_Team 2014) based Gower’s coefficient of similarity (Gower 1971) in *daisy* and with complete linkage in *hclust* (Fig. 3). After sampling was done for this study, eight koalas developed cancer (primarily lymphoma or leukemia) and another six koalas died of natural causes (age-related). To determine if there were any associations between developing cancer and MHC alleles, both combined haplotype and individual allele prevalence were examined in this subset of deceased koalas. While there was no significant difference detected in the overall MHC haplotypes of these koalas (*χ*^2^ = 23.946, df = 29, *p* = 0.7316) (graphically seen in the lack of clustering by causes of death in Fig. 3), allele DBB*03 was significantly more prevalent in koalas that developed cancer (5/8; 63%) than koalas that died of natural causes (0/6; 0%) (Fisher exact *p* = 0.031). It should be acknowledged that association of an MHC allele with neoplasia provides no evidence of causation, as this outcome could be related to another linked genetic or retroviral trait. As the sample size in this analysis was relatively small, monitoring will continue in these koalas and reanalysis will be undertaken when sample sizes are larger.

**Fig. 3:**
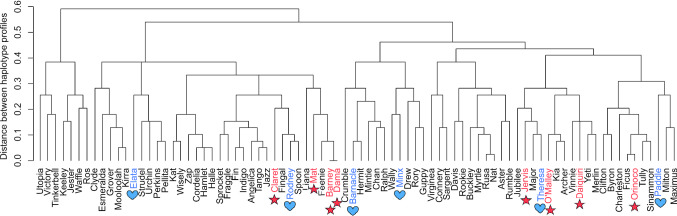
MHC haplotype clustering of captive koalas in this study. Koalas that developed cancer (primarily lymphomas and leukemias) are indicated in red with red stars, while koalas that died from natural causes (related to old age) are indicated in blue with blue hearts

In conclusion, this study designed and tested a high-throughput protocol to determine the MHC allelic profile of koala classic class I and class II beta subfamily genes. Using established PCR primer sets, standard Illumina paired end sequencing and freely available software, this method resulted in 96% congruence of allele assignment within 36 koala family units over six MHC loci. Alleles detected in this study expanded the list of known koala MHC alleles, and an association between the presence of DBB*03 and koalas developing cancer was detected. This protocol offers a reliable method for expanded study in the important area of koala immunogenetics.
